# Dry preserved multilayered fibroblast cell sheets are a new manageable tool for regenerative medicine to promote wound healing

**DOI:** 10.1038/s41598-022-16345-6

**Published:** 2022-07-22

**Authors:** Yutaro Matsuno, Masashi Yanagihara, Koji Ueno, Toshiro Saito, Hiroshi Kurazumi, Ryo Suzuki, Shunsaku Katsura, Atsunori Oga, Kimikazu Hamano

**Affiliations:** 1 Department of Surgery and Clinical Science, Yamaguchi University Graduate School of Medicine, Ube, Japan; 2 Department of Molecular Pathology, Yamaguchi University Graduate School of Medicine, Ube, Japan

**Keywords:** Cell therapies, Drug safety

## Abstract

This study investigated the therapeutic effects of dry-preserved multi-layered fibroblast cell sheets (dry sheets) on cutaneous ulcers. Dry sheets were prepared by air-drying multi-layered fibroblast cell sheets (living sheets) to cease their life activities. Before in vivo application, we tested the release of growth factors into the medium to examine the mechanisms of dry sheets in wound healing. Vascular endothelial growth factor (VEGF) and hepatocyte growth factor (HGF) were released from both dry and living sheets, while high levels of fibroblast growth factor-2 (FGF-2) and high mobility group box 1 (HMGB1) protein were only from dry sheets. An in vitro fibroblast proliferation assay revealed that the dry sheet eluate significantly enhanced cell proliferation and VEGF and HGF production compared with living sheet eluate. FGF-2-neutralizing antibodies significantly blocked this proliferative response. In wounds created on diabetic mice, the dry sheet-treatment groups using autologous or allogeneic cells showed significantly accelerated wound closure compared with that in the no-treatment group. The storage stability of the dry sheet was better at refrigeration temperature than at room temperature and remained stable for at least 4 weeks. Our data indicated that allogeneic dry sheets represent a promising new tool for regenerative medicine that promotes wound healing.

## Introduction

Cell sheet technology has been recently developed to improve the retention of transplanted cells in the grafted region^1^, and cell sheet transplantation therapy has been used to prevent various diseases and postoperative complications, including ischemic cardiomyopathy, air leakage after lung operations, and stenosis after endoscopic submucosal dissection of the esophagus^2–4^. This technique can also be used to treat cutaneous ulcers.

Previously, we developed a mixed cell sheet consisting of fibroblasts and peripheral blood mononuclear cells (PBMNCs), which increased the vascular endothelial growth factor (VEGF) secretion from fibroblasts by the synergistic effects of the co-cultured with PBMNCs and the treatment of hypoxic preconditioning^5,6^. One of the main mechanisms of action for the therapeutic effects of cell sheets involves a paracrine effect due to the production of growth factors and cytokines from the cell sheets. Mixed cell sheets have been shown to have success in wound healing for mouse and rabbit skin ulcer models^5,6^. In our previous work, we developed a simple method to manufacture multi-layered cell sheets and reported the usefulness of autologous multi-layered mixed sheets, which were treated with hypoxic preconditioning, in a mouse skin ulcer model^7^. Further, in a clinical study in which a hypoxic preconditioned autologous multi-layered mixed cell sheet containing PBMNC**s** and human oral mucosal fibroblast cells was transplanted onto a leg ulcer, these safety and wound healing promoting effects were confirmed^8^. However, three of the six cases did not result in transplantation: one due to a lack of fibroblast cell growth, while the other two did not meet the criteria for transplantation due to poor VEGF production^8^. This clinical study using autologous cells also revealed a high rate of poor patient fibroblast performance. Cell sheet therapy using autologous cells is a promising method with the potential to successfully treat patients, necessitating a stable supply of cell sheets prepared using allogeneic cells. The purpose of autologous PBMNCs in the mixed cell sheets was to improve the secretory capacity of the fibroblasts. However, layering of cell sheets sufficiently enhanced the function of fibroblast sheets, and there was little difference between the effects of fibroblasts alone and mixed cell sheets in a mouse skin ulcer model^7^. We confirmed that sufficient growth factors were secreted by the multi-layered fibroblast cell sheets and that the wound-healing effect of allogeneic multi-layered fibroblast cell sheets was comparable to that of autologous cell sheets in a mouse ulcer model. Although some local immunity occurs, this does not adversely affect wound healing^9^. Thus, when considering the clinical application of cell sheet transplantation therapy, cell sheets prepared from allogeneic cells that can be stably supplied may represent the best approach.

To improve the convenience and uptake of cell sheet therapy, it will be essential to develop a simple cell sheet preservation method that can be used quickly. One option is the use of extracellular matrix (ECM) sheets, which are decellularized by freeze-thaw^10^. These sheets have the advantages of storage stability and lower rates of immune rejection and act as a scaffold for cells when transplanted^10,11^. However, they retain little bioactive substances due to cell damage caused by freeze–thaw. Otherwise, dry preservation is the optimal method in terms of convenience. Studies on lyophilized epidermal cell sheets^12,13^ and amniotic membrane sheets^14,15^ have been conducted; however, there are no reports yet on dry-preserved fibroblast sheets. Therefore, in this study, we aimed to develop a dry-preserved multi-layered fibroblast cell sheet (dry sheet) and assess its therapeutic effects using allogeneic fibroblasts in a mouse cutaneous ulcer model. To verify the effectiveness of the dry sheets, we compared them to freeze–thaw sheets (FT sheets), which contain almost no cell contents after repeated freeze–thaw cycles, and multi-layered fibroblast cell sheets (living sheets). All cell sheets, living, dry, and FT sheets used in this study were produced from hypoxic preconditioned multi-layered fibroblast cell sheets, which have been reported previously^7^, since hypoxic preconditioning is effective in increasing the secretory potency of the fibroblasts^5,6^. Prior to in vivo application, the release of growth factors into the medium was tested to study the action mechanisms of dry sheets in wound healing. The dry sheets are not expected to secrete angiogenic factors such as VEGF and hepatocyte growth factor (HGF), which are secreted by living sheets. However, drying may cause damage to cell membranes and the release of intracellular substances upon rehydration. In this study, we focused on fibroblast growth factor-2 (FGF-2)^16–20^ and high mobility group box 1 (HMGB1) protein^21–26^, which are released from damaged cells and are involved in wound healing. We investigated whether these growth factors are released from the dry sheets into the medium and whether they retain their biological activities.

## Results

### Preparation of dry-preserved multi-layered fibroblast cell sheets

**Figure 1:**
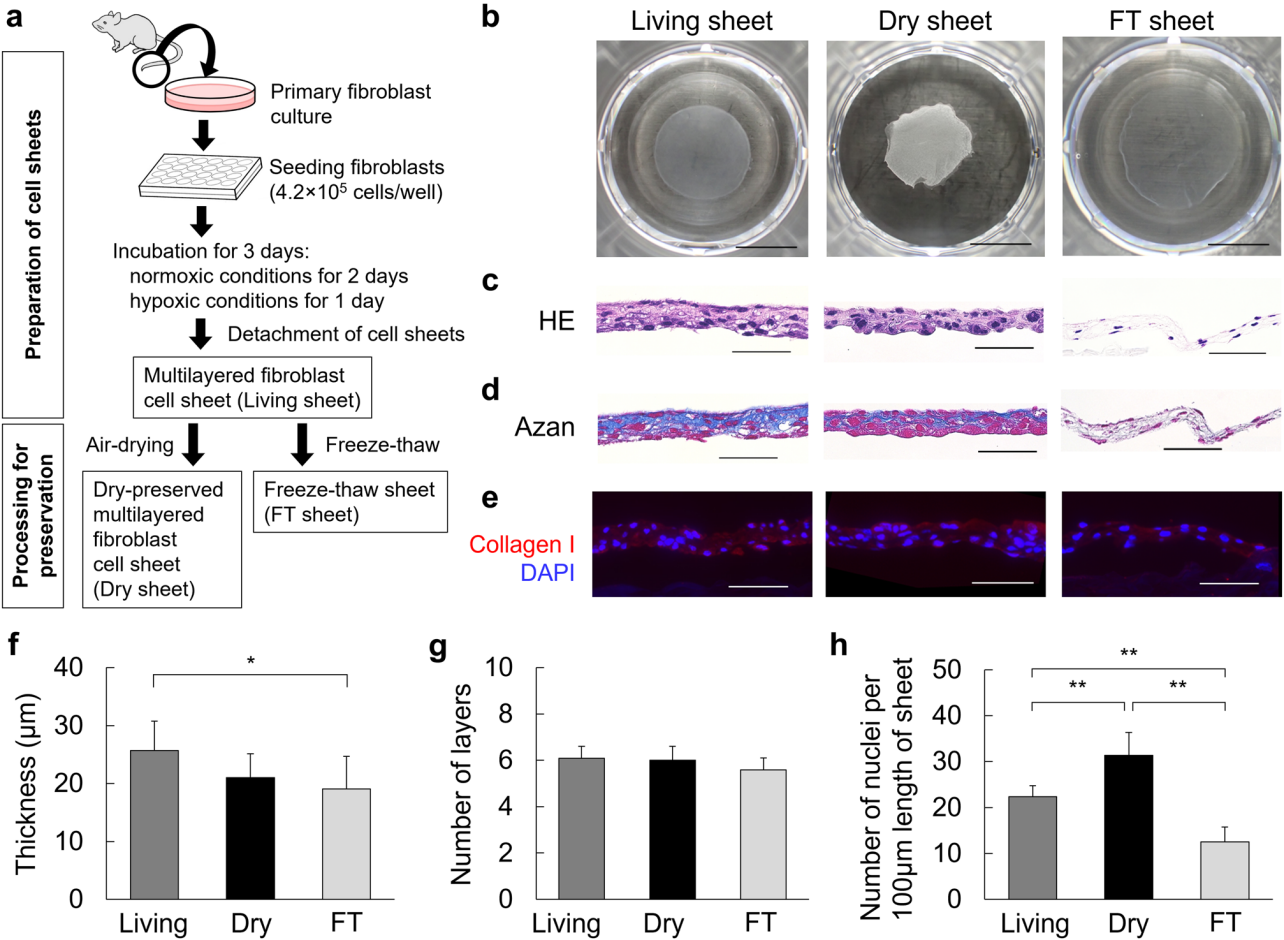
Preparation and histological analysis of cell sheets. (**a**) Schematic diagram of manufacturing multi-layered fibroblast cell sheets, dry-preserved multi-layered fibroblast cell sheets, and freeze–thaw (FT) sheets. (**b**) Morphological observations of the living, dry, and FT sheets. Each cell sheet was placed in a well of the 24-well plate (scale bar = 5 mm). (**c**) Cross sections of the living, dry, and FT sheets with HE staining (scale bar = 50 µm). (**d**) Cross sections of the living, dry, and FT sheets with Azan staining (scale bar = 50 µm). (**e**) Cross-sections of the living, dry, and FT sheets with immunofluorescent staining of collagen type I (red) and DAPI (blue) (scale bar = 50 µm). (**f**) Average thickness of each cell sheet. Values are expressed as mean ± SD (*P < 0.05, Tukey–Kramer test, n = 6 per group). (**g**) Average number of layers in each cell sheet. Values are expressed as mean ± SD (Tukey–Kramer test, n = 6 per group). (**h**) Average number of nuclei in each cell sheet. The number of nuclei within 100 μm along the long axis was measured in sections of each cell sheet. Values are expressed as mean ± SD (**P < 0.01, Tukey–Kramer test, n = 6 per group). Living: living sheets; Dry: dry sheets; FT: freeze-thawed sheets.

Cultured primary fibroblasts, derived from mouse tail skin (C57BL/6N), were seeded at 4.2 × 10^5^ cells per well in normal 24-well culture plates to form a multi-layered cell sheet, and then cells were incubated under normoxic conditions (37 °C, 5% CO_2_, 20% O_2_) for 2 days, followed by hypoxic conditions (33 °C, 5% CO_2_, 2% O_2_) for 1 day for treatment of hypoxic preconditioning^5–7^. After incubation, the multi-layered fibroblast cell sheets were gently detached from the culture plates after dispase treatment for use as living sheets in this study. As shown in Fig. 1a, both dry sheets and FT sheets were produced from living sheets by air-drying for 30 min or freeze–thaw cycles, respectively. Further details are provided in the “Material and methods” section. The morphological observations of each cell sheet are shown in Fig. 1b. The living sheet shrank to approximately half of its attached size after detachment from the culture plates. The dry sheet was hard enough to be grasped by tweezers, yet maintained its structure (Supplementary Fig. S1). The FT sheet was more transparent than the living sheet.

### Histological findings of cell sheets

Cross-sections of each sheet (i.e., living, dry, or FT) stained with hematoxylin–eosin (HE) and Azan are shown in Fig. 1c,d. All sheets were multi-cell layered. The dry sheet (21 ± 4.2 µm) was slightly thinner than the living sheet (25.7 ± 5.1 µm), and had five to seven layers (Fig. 1f,g). On the dry sheet, there were slight morphological changes in the cell nuclei, which swelled and became more variable in size. The number of nuclei per 100 μm length of the sheet was significantly higher on the dry sheet, probably because of the smaller volume after drying (Fig. 1h). The changes were relatively large on the FT sheet, in which the number of nuclei and chromatin were notably reduced. Both blue areas (indicating collagen by Azan staining) and red areas (indicating ECM type I collagen, as revealed by fluorescent immunostaining) were found on all three sheets; their prominence was ranked in the order of living, dry, and FT sheets (see Fig. 1e).

### Characteristics of dry-preserved multi-layered fibroblast cell sheets

**Figure 2:**
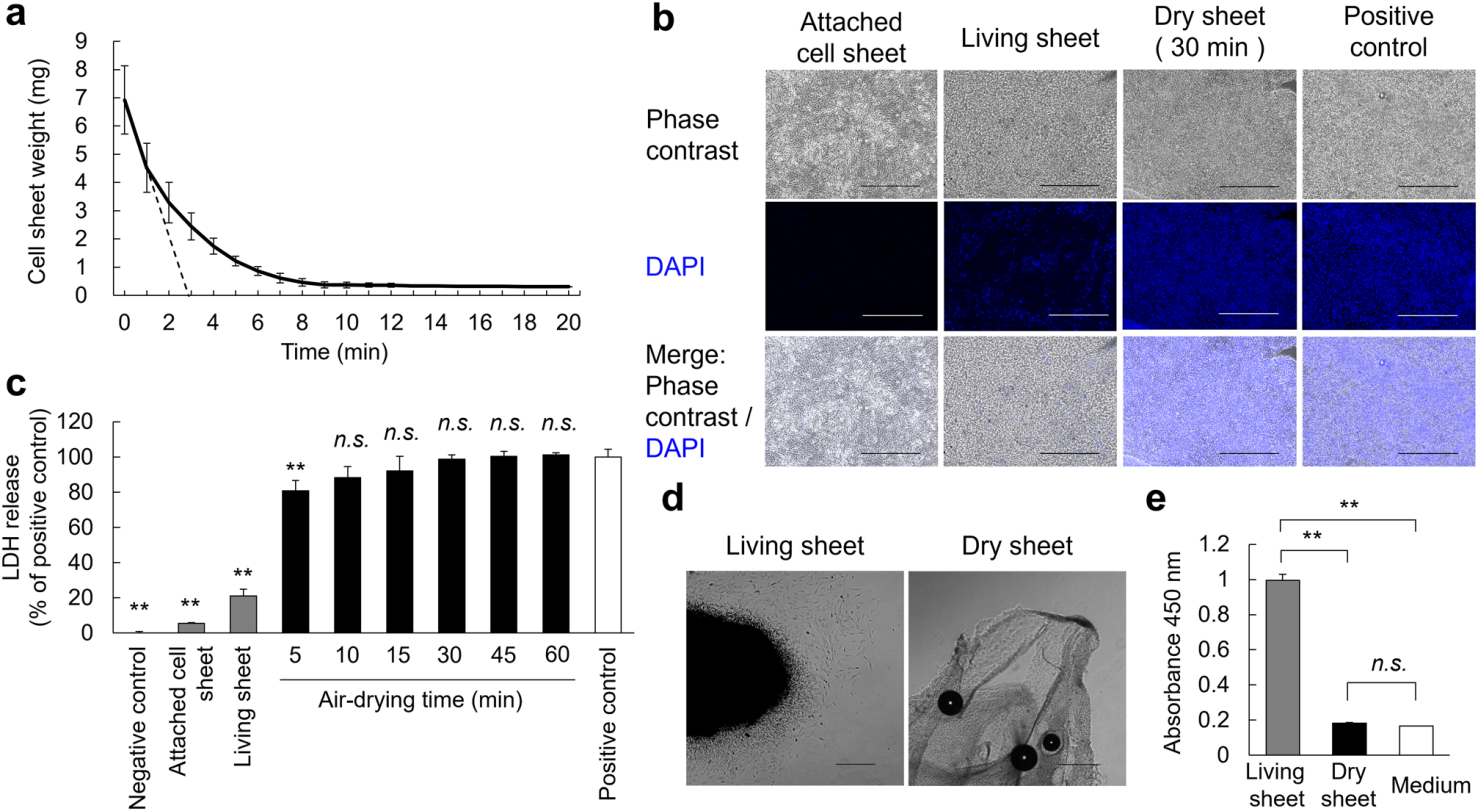
Investigation of weight change and cytotoxicity of cell sheets with drying time. (**a**) Changes in the weight of a multi-layered fibroblast cell sheet during drying time on the bio-clean bench. The average weight of a living sheet was 6.9 mg. While drying, the average weight was 0.31 mg after an average of 13.7 min. Three independent experiments were performed. The solid and the dotted lines show the weight and the initial drying rate, respectively. (**b**) DAPI staining of unfixed living and dry sheets. Living sheets immersed in methanol were used as the positive control and attached cell sheets as the negative control (scale bar = 50 µm). (**c**) Evaluation of the relationship between cell sheets drying time and cell membrane damage by LDH release assay using the living sheet immersed in Lysis Buffer as the positive control and the PBS as the negative control. Values are expressed as mean ± SD (**P < 0.01, n.s.: not significant versus positive control, Tukey–Kramer test, n = 6 per group). (**d**) Observation of the adhesive ability of cell sheets after 24 h of re-culture. The living sheets (left) adhered to the culture dish, and fibroblasts were observed to migrate from the edges, but not in dry sheets (right; scale bar = 50 µm). (**e**) Evaluation of the cell metabolic activity of a 24 h re-cultured cell sheet by WST-8 reagent. Values are expressed as mean ± SD (**P < 0.01, n.s.: not significant, Tukey–Kramer test, n = 4 per group). Medium: CTS AIM-V + 10% FBS.

Cell sheets were airdried on a bio-clean bench under the following conditions: a temperature of 30.7 °C (ranging from 27.6 to 31.4 °C), humidity of 39.6% (ranging from 31.1 to 49.6%), and wind speed ranging from 0.1 to 0.4 m/s. The weight change from the living sheet to the dry sheet during air drying was measured. The average weight of the living sheet was 6.9 mg. Air-drying decreased the water content of the cell sheet, and the weight of the cell sheet reached equilibrium at an average of 13.7 min (9, 12, and 20 min) from three independent experiments. The average weight of a dry sheet that reached equilibrium was 0.31 mg (Fig. 2a). The average surface area of the dry sheet was 0.31 cm^2^/sheet, and the moisture content of the dry sheet, measured by the Karl Fischer method, was 3.2%. The initial drying rate was 7.9 mg/min·cm^2^.

### Multi-layered fibroblast cell sheets cease vital activity as cell membranes are damaged by drying

To evaluate cell death due to drying, unfixed cell sheets were stained with 4′,6-diamidino-2-phenylindole (DAPI) to detect cell membrane damage. Only some of the nuclei were stained with DAPI in the living sheets (Fig. 2b). At 5, 10, and 15 min of drying, the nuclei of cell sheets were stained with DAPI from the cell sheet periphery, but there were unstained areas in the center of the cell sheets (Supplementary Fig. S2). Almost all nuclei of the sheets dried for 30 min were stained with DAPI (Fig. 2b). This observation suggests that the permeability of the cell membrane was affected by the drying process. To quantitatively evaluate damaged cell membranes, a lactate dehydrogenase (LDH) release assay was performed to investigate the relationship between the drying time and the proportion of damaged cells. Compared with the positive control (the living sheet treated with lysis buffer), the LDH release rate was 21% in the living sheet. The rate of release of LDH increased with the duration of drying and was 88% at 10 min, 92% at 15 min, and ≥ 98% after 30 min, corresponding to the attainment of an equilibrium dry weight (Fig. 2c).

To confirm cell survival, the cell sheets were re-cultured for 24 h. The living sheet adhered to the bottom surface of the culture dish and fibroblasts migrated from the margin of the sheet, whereas in the dry sheet, there was no adhesion to the culture dish or migration of fibroblasts (Fig. 2d). Dry cell sheets cultured for 24 h exhibited no metabolic activity, and the absorbance was comparable to that of the culture medium (Fig. 2e). These results demonstrate that the cell membranes on the dry sheet were considerably weakened by drying and that drying the sheet irreversibly ceased life activity, reflecting cell death.

Taking these observations together, the drying time was set to 30 min to ensure reliable drying conditions. Therefore, the following experiments were performed using a dry sheet that was air-dried for 30 min and stored at room temperature (23 °C) for up to 1 week.

### Growth factors and cytokines in dry sheets are released from cells

**Figure 3:**
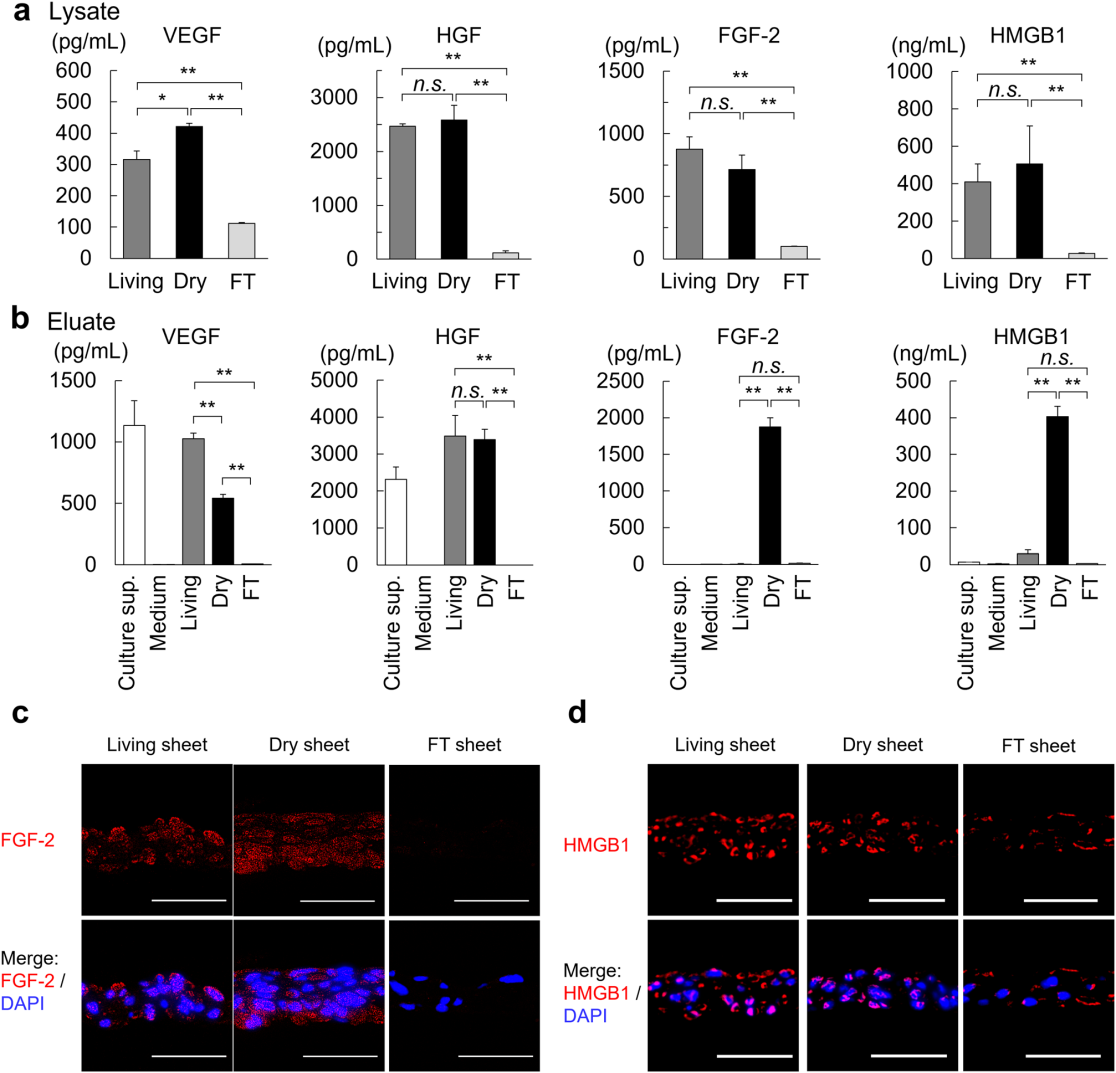
Growth factors and cytokines in dry sheet released from cells. (**a**) The retention level of growth factors from each cell sheet. The concentration of VEGF, HGF, FGF-2 and HMGB1 of the supernatant of each cell sheet lysate as measured by ELISA. The lysate was prepared by immersing cell sheet in 200 µL cell lysis buffer 2. Values are expressed as mean ± SD (**P < 0.01, n.s.: not significant, Tukey–Kramer test, n = 3 per group). (**b**) Release amounts of growth factors from each cell sheet. Concentrations of VEGF, HGF, FGF-2 and HMGB1 of the supernatant of eluate samples as measured by ELISA. The eluate samples were prepared by immersing each cell sheet in 200 µL CTS AIM-V with 10% FBS for 24 h in normoxic conditions. Values are expressed as mean ± SD (**P < 0.01, Tukey–Kramer test, n = 4 per group). Significant differences were examined only between living, dry, and FT sheets. Culture sup.: Culture supernatant at the time of sheet preparation (CTS AIM-V + HFDM-1(+) + 5% FBS), Medium: CTS AIM-V + 10% FBS. (**c**) Cross-sections of the living, dry, and FT sheets with immunofluorescence staining of FGF-2 (red) and DAPI (blue); (scale bar = 50 µm). (**d**) Cross-sections of the living, dry, and FT sheets with immunofluorescence staining of HMGB1 (red) and DAPI (blue; scale bar = 50 µm). Living: living sheets; Dry: dry sheets; FT: freeze-thawed sheets.

To assess the growth factors and cytokines remaining in dry sheets using the supernatants of lysed cell sheets, we measured the levels of VEGF, HGF, FGF-2, and HMGB1 using ELISA. In dry sheets, the amount of each growth factor was equivalent to that in living sheets, whereas FT sheets showed significantly lower values (Fig. 3a). We subsequently examined whether these growth factors were released from dry sheets into the medium. Each cell sheet was immersed in 200 µL medium and incubated for 24 h, followed by the collection of supernatants from the immersed cell sheet. VEGF and HGF were detected in both the living sheet and dry sheet eluates, whereas FGF-2 and HMGB1 were detected only in the dry sheet eluate (Fig. 3b). We further investigated the localization of FGF-2 and HMGB1 in each cell sheet by indirect immunofluorescence staining. FGF-2 was localized mainly in the cytoplasm of living and dry sheets but was not detected in the FT sheets (Fig. 3c). HMGB1 was found to be mainly colocalized at the cell nucleus in living and dry sheets but was not detected in FT sheets (Fig. 3d). In addition, immunostaining signals for FGF-2 and HMGB1 were markedly decreased in the dry sheets after immersion (Supplementary Fig. S3). These observations demonstrate that intracellular FGF-2 and HMGB1 in dry sheets were readily released from cells because the cell membrane and nuclear envelope were weakened by air drying.

### Enhanced cell proliferation and VEGF and HGF production in fibroblasts by the eluate of dry sheets

**Figure 4:**
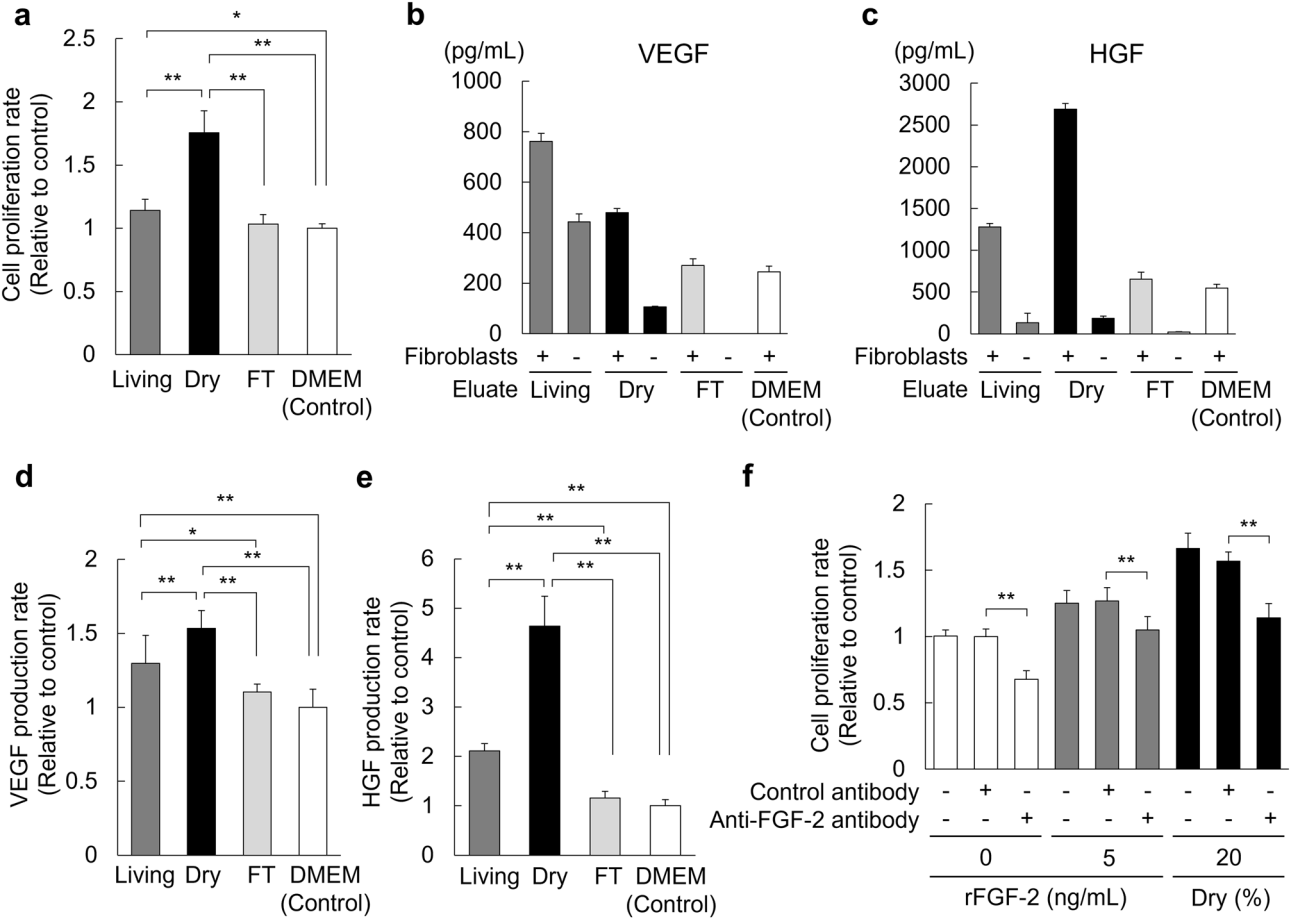
Enhanced cell proliferation, VEGF and HGF production in fibroblasts by the eluate of dry sheets. Eluate samples were prepared by immersing each cell sheet in 200 μL DMEM for 24 h, and the supernatant was collected after centrifugation. (**a**) Fibroblasts were seeded in 96-well plates at a concentration of 8000 cells per well in 100 μL DMEM with 10% FBS, followed by the addition of 100 μL of the eluate samples or DMEM as a control. In addition, 100 μL the eluate sample was added to 100 μL DMEM with 10% FBS per well as an eluate sample without fibroblasts. After 48 h of incubation under normoxic conditions, the culture supernatant was collected and the cell proliferation assay by WST-8 reagent was performed. The cell proliferation rate was calculated using fibroblasts cultured in DMEM as the control. (**b**,**c**) Concentrations of VEGF and HGF in the culture supernatant of fibroblasts stimulated by eluate. (**d**,**e**) VEGF and HGF production ratios from fibroblasts upon eluate stimulation. Ratios were calculated using the following formula: [(supernatant of fibroblasts cultured with the eluate sample) − (supernatant of the eluate sample without fibroblasts)]/(supernatant of fibroblasts cultured with DMEM). Fibroblasts cultured in DMEM were used as the control. (**f**) The eluate samples were incubated for 60 min at 37 °C with anti-FGF-2 or control antibody. Fibroblasts were seeded in 96-well plates at a concentration of 8000 cells per well in 100 μL DMEM with 1% FBS, followed by the addition of 100 μL of either the eluate sample with the antibodies, or DMEM containing rFGF-2 (0 or 5 ng/mL) with antibodies. After a 48 h incubation under normoxic conditions, the culture supernatant was aspirated and the cell proliferation assay by WST-8 reagent was performed. The cell proliferation rate was calculated using fibroblasts cultured in 0 ng/mL rFGF-2 with the control antibody as the control. Data in all panels are expressed as mean ± SD (*P < 0.05, **P < 0.01, Tukey–Kramer test, n = 9 per group). Living: living sheet eluate, Dry: dry sheets eluate, FT: freeze-thawed sheets eluate, rFGF-2: recombinant FGF-2 protein.

To investigate the bioactivity of the dry sheet eluate, we examined cell proliferation and VEGF and HGF production in fibroblasts in vitro. Fibroblasts were cultured for 48 h with medium and eluate mixed at a 1:1 ratio. The dry sheet eluate was 1.75 times higher than the control and showed significantly higher cell proliferation than the living or FT sheet eluate (Fig. 4a). Next, the concentrations of VEGF and HGF in culture supernatants were measured (Fig. 4b,c). Significant amounts of VEGF and HGF were detected in the dry eluate with fibroblasts compared with those in the control, whereas high levels of VEGF and HGF were not detected in the dry eluate without fibroblasts, as seen in Fig. 3b. This could be because of the stability of the growth factors during 48 h of incubation. Therefore, the differences between values for eluate with and without fibroblasts was considered as the VEGF and HGF contents newly produced from fibroblasts owing to the stimulation of the eluate and compared as a ratio to the control (Fig. 4d,e). The dry sheet eluate induced significantly higher VEGF and HGF production rates at 1.53 and 4.64 times those in the control. These results demonstrate that the dry sheet eluate had biological effects on cells.

### FGF-2 neutralizing antibody prevents the proliferation of fibroblasts stimulated by the eluate of dry sheets

FGF-2 is a potent growth factor. The dry sheet eluate contained a large amount of FGF-2, suggesting FGF-2 may affect biological activity. Therefore, we examined whether FGF-2 in the eluate directly affected fibroblast proliferation using a neutralizing antibody against FGF-2 or recombinant FGF-2 protein (rFGF-2). Under the presence of a control antibody (Mouse IgG1 isotype control), the proliferative response of fibroblasts was promoted by dry sheet eluate or rFGF-2. However, these proliferative responses were significantly inhibited by FGF-2 neutralization (Fig. 4f). These results indicate that the biological activity of the dry sheets was mainly due to the effect of FGF-2.

### Therapeutic effect of dry sheets in a mouse cutaneous wound model

**Figure 5:**
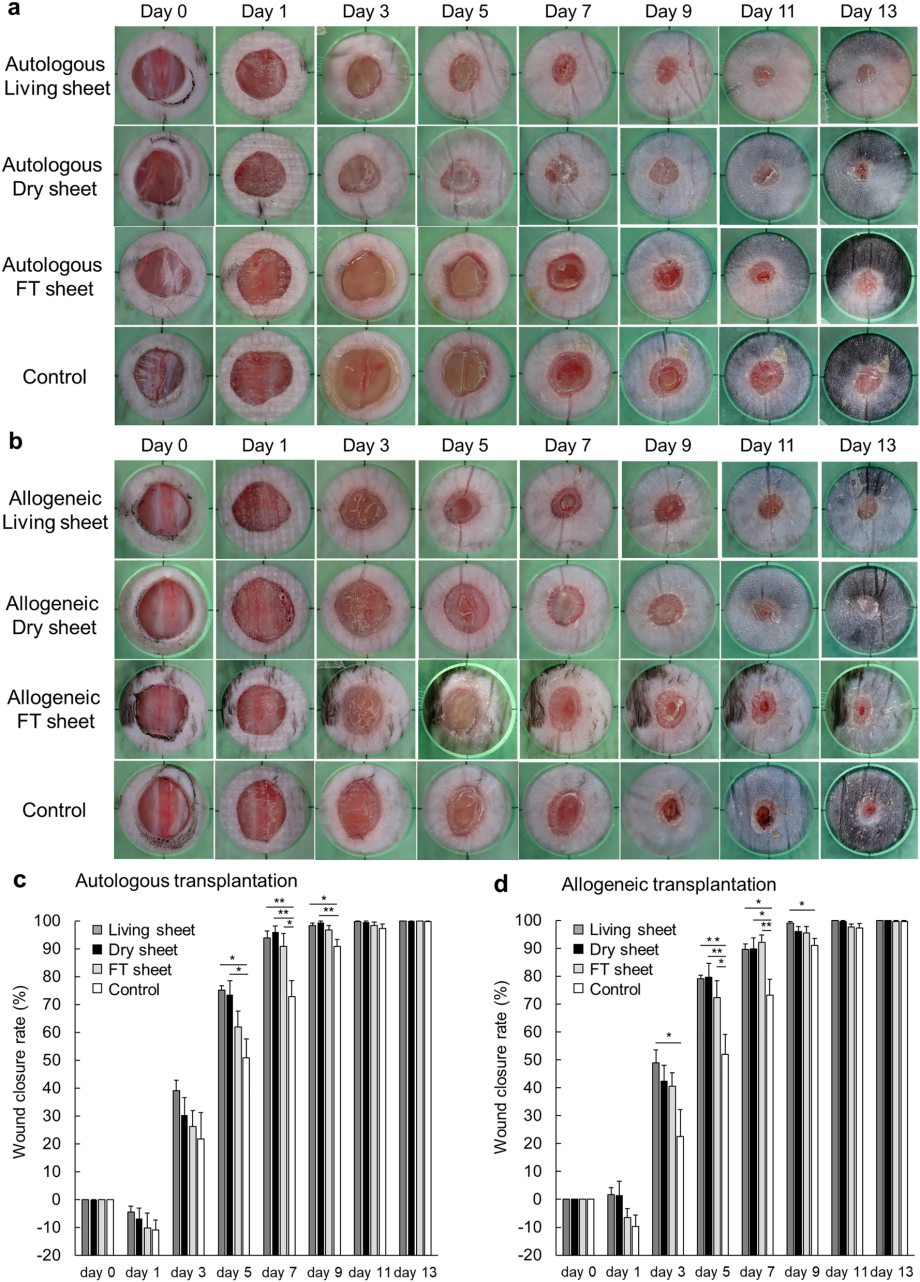
Therapeutic effect of dry-preserved multi-layered fibroblast cell sheets. (**a**) Representative macroscopic images of the wound on days 0, 1,3, 5, 7, 9, 11, and 13 in the autologous cell sheets transplantation therapy. Living sheets, dry sheets, and FT sheets group, which were prepared by autologous fibroblasts (C57BL/6N mouse), were transferred onto skin defects in 6 mm dorsal full-thickness cutaneous wound closure models of diabetic male C57BL/6N mice. Control indicates no treatment. (**b**) Representative macroscopic images of the wound on days 0, 1, 3, 5, 7, 9, 11, and 13. in the allogeneic cell sheets transplantation therapy. Living sheets, dry sheets, and FT sheets group, which were prepared by allogeneic fibroblasts (C3H/He mouse) were transferred onto skin defects in 6 mm dorsal full-thickness cutaneous wound closure models of diabetic male C57BL/6N mice. Control indicates no treatment. (**c**) The wound closure rate of the autologous cell sheet transplantation therapy by living sheets (n = 6), dry sheets (n = 6), FT sheets (n = 6) and control (no-treatment) group (n = 6). The wound closure rate was calculated as the percentage of initial wound area at the indicated time points. Error bars indicate standard error. Values are expressed as mean ± SE (*P < 0.05, **P < 0.01, Tukey–Kramer test). (**d**) The wound closure rate of the allogeneic cell sheet transplantation therapy by living sheets (n = 6), dry sheets (n = 6), FT sheets (n = 6) and control (no-treatment) group (n = 6). The wound closure rate was calculated as the percentage of initial wound area at the indicated time points. Error bars indicate standard error. Values are expressed as mean ± SE (*P < 0.05, **P < 0.01, Tukey–Kramer test).

Autologous (C57BL/6N mouse) and allogeneic (C3H/He mouse) cell sheets were transplanted into 6 mm full-thickness dorsal skin defects in diabetic mice (male, C57BL/6N), and each wound was evaluated on days 0, 1, 3, 5, 7, 9, 11 and 13 (n = 6; Fig. 5a,b). The rate of wound closure was significantly higher in the autologous and allogeneic dry sheet treatment groups than in the no-treatment control group on day 5 (autologous dry sheet and allogeneic dry sheet group vs. no-treatment control group: 74.2 ± 5.0% [*P* < 0.05] and 80.0 ± 4.9% [*P* < 0.01] vs. 51.0 ± 7.2%), on day 7 (autologous dry sheet and allogeneic dry sheet vs. no-treatment: 95.8 ± 2.1% [*P* < 0.01] and 90.0% ± 4.0% [*P* < 0.05] vs. 72.4 ± 5.7%), and on day 9 (autologous dry sheet vs. no-treatment: 99.4 ± 0.5% [*P* < 0.01] vs. 91.0 ± 2.4%; Fig. 5c,d).

Observation of tissue specimens obtained 30 days after transplantation of each cell sheet showed no abnormal findings in the tissue after wound healing in all cell sheet transplantation groups, both using autologous and allogeneic cells, compared to the no-treatment group (Supplementary Fig. S4).

### Positioning of the allogeneic dry sheets during the wound healing process

**Figure 6:**
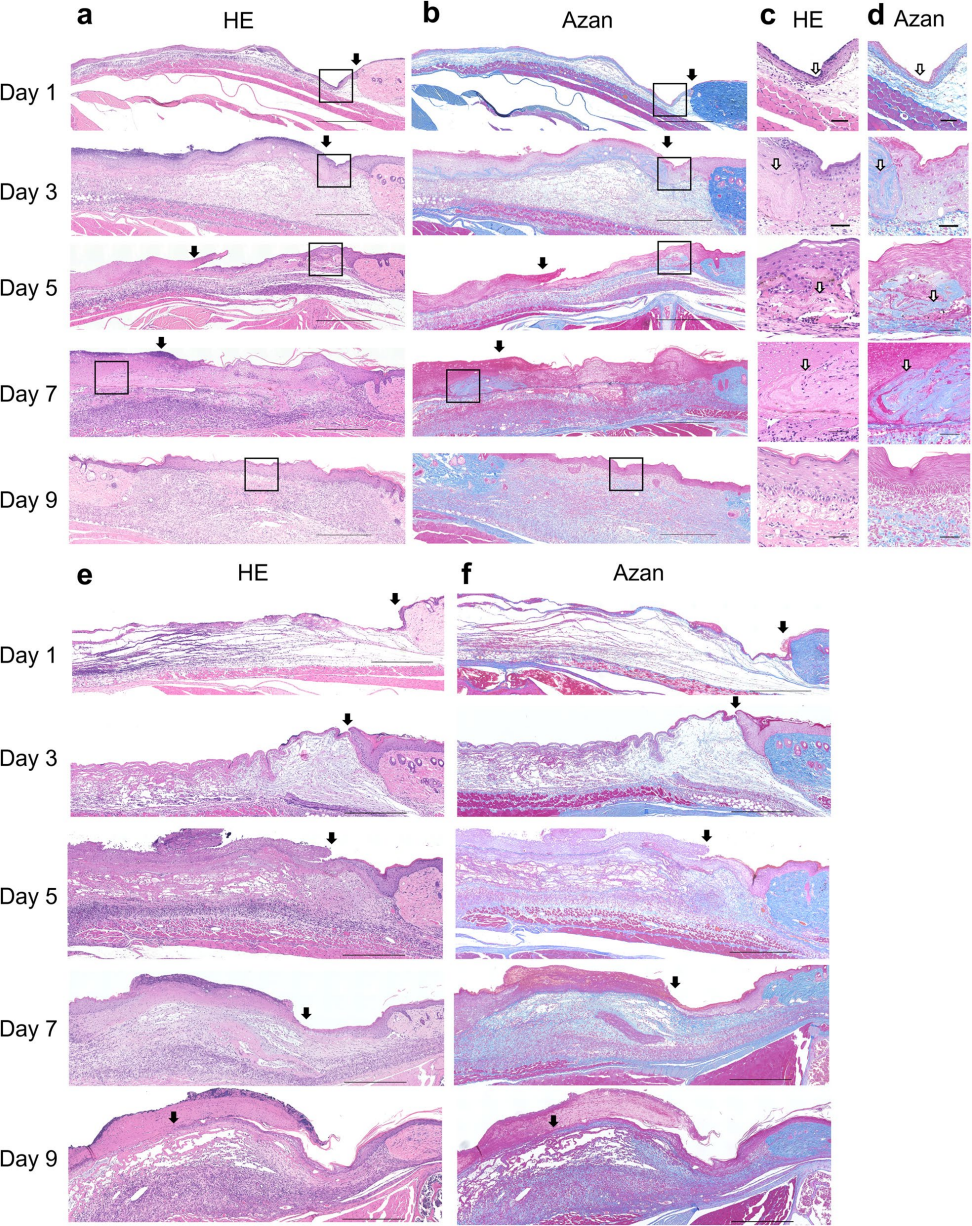
Histological analysis of treatment course after dry sheets transplantation. (**a**) HE-stained cross section of a mouse cutaneous wound transplanted with the dry sheet on days 1, 3, 5, 7, and 9 (scale bar = 500 µm). The black arrows show the neo-epithelium edge. (**b**) Azan-stained cross section of a mouse cutaneous wound transplanted with the dry sheet on days 1, 3, 5, 7 and 9 (scale bar = 500 µm). The black arrows show the neo-epithelium edge. (**c**) The image enclosed in the square in (**a**) on days 1, 3, 5, 7 and 9 (scale bar = 50 µm). The white arrows show the dry sheet. (**d**) The image enclosed in the square in (**b**) on days 1, 3, 5, 7 and 9 (scale bar = 50 µm). The white arrows show the dry sheet. HE: hematoxylin–eosin. (**e**) HE-stained cross section of skin wound on mice in the no treatment group on days 1, 3, 5, 7 and 9 (scale bar = 500 µm). The black arrows show the neo-epithelium edge. (**f**) Azan-stained cross section of skin wound on mice in the no treatment group on days 1, 3, 5, 7 and 9 (scale bar = 500 µm). The black arrows show the neo-epithelium edge.

We investigated the position of allogeneic dry sheets during wound healing. The dry sheets were discriminated against the blue areas at the site of application (Fig. 6b,d) because the collagen fibers of the sheets were stained blue by Azan staining (Fig. 1d). One day after transplantation, a dry sheet covered the entire wound (Fig. 6a,c). The dry sheets were infiltrated with leukocytes, which appeared to be neutrophils or macrophages. There was a clear difference in the nucleus color between the infiltrated leukocytes and the sheet. The nuclei of infiltrated leukocytes were clearly visible using HE staining, whereas the nuclei of the dry sheet-derived cells stained faintly and indistinctly with time after transplantation (Supplementary Fig. S5). At 3, 5, and 7 days after transplantation of dry sheets, Azan staining showed “folded” sheet-like structures and collapse compared to 1 day after transplantation. The dry sheet was present directly under the formed scab or epidermis, and it could not be confirmed from cross-section on day 9. In contrast, no sheet-like structures were observed in the no-treatment control group (Fig. 6e,f).

Interestingly, histological observations indicated that dry sheet treatment promoted wound healing. On day 3 after transplantation, the allogeneic dry-sheet treatment group showed a significant increase in the number of keratinocyte layers and microvessels at the wound edge compared with that in the no-treatment group (Supplementary Fig. S6a–d). In accordance with macroscopic observations of wound healing (Fig. 5b,d), the neo-epithelium length on days 5 and 7 after transplantation showed a significant prolongation in the allogeneic dry-sheet treatment group compared with that in the no-treatment control group (Supplementary Fig. S6e–h).

### Storage stability of dry sheets

**Figure 7:**
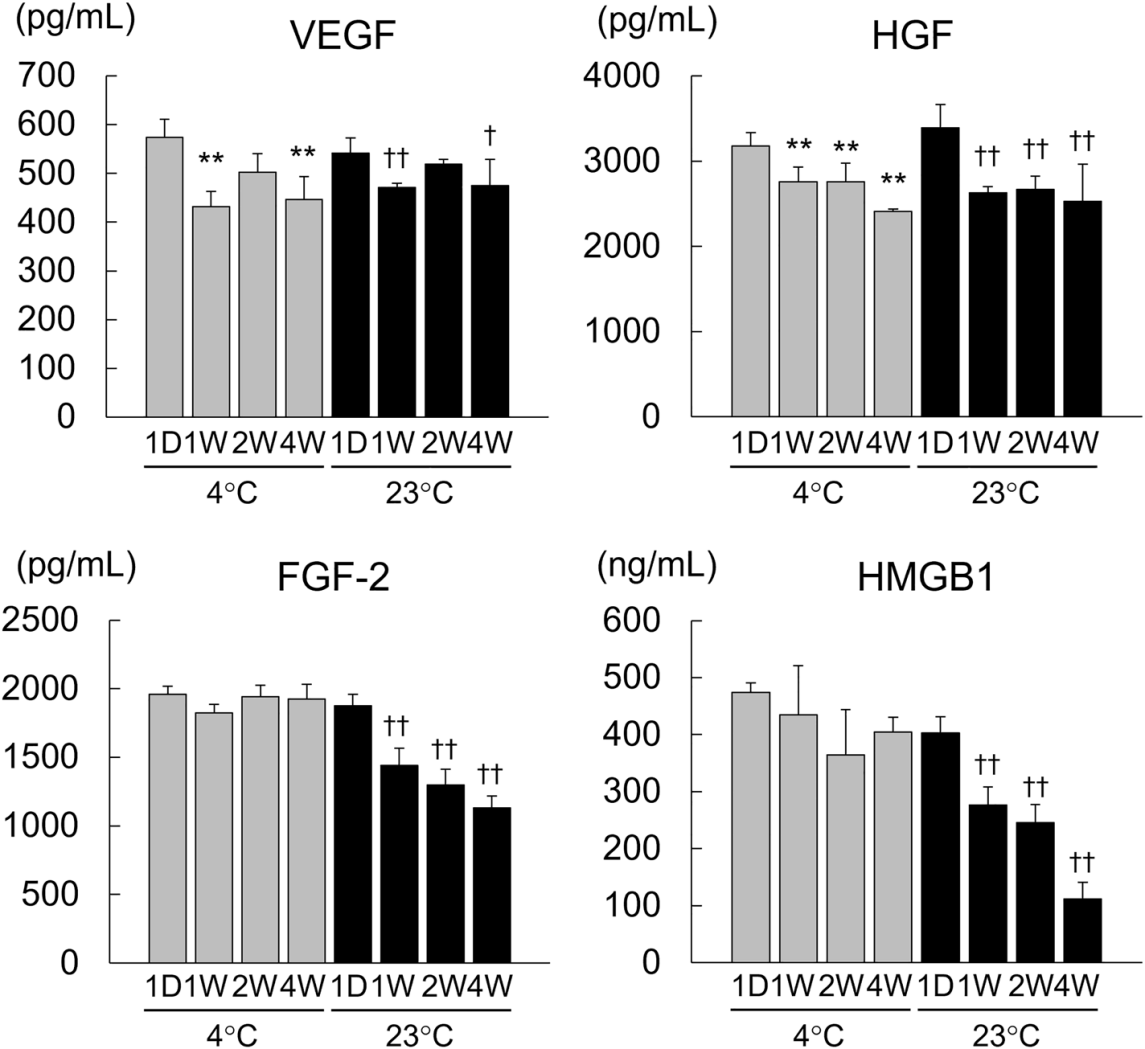
Investigation of the storage stability of dry sheets. Simultaneously prepared dry sheets were stored at refrigeration temperature (4 °C) and room temperature (23 °C) for either 1 day, 1, 2, or 4 weeks, and then the eluates were prepared by immersing each dry sheet in 200 µL CTS AIM-V with 10% FBS for 24 h under normoxic conditions (37 °C, 5% CO_2_, 20% O_2_) and stored at − 30 °C until measurement. The concentrations of VEGF, HGF, FGF-2, and HMGB1 in the supernatant of the eluate samples were measured by ELISA. 1D: Storage period of 1 day. 1 W: Storage period of 1 week. 2 W: Storage period of 2 weeks. 4 W: Storage period of 4 weeks. Values are expressed as mean ± SD (*P < 0.05 versus 1D 4 ℃, **P < 0.01 versus 1D 4 ℃, ^†^P < 0.05 versus 1D 23 ℃, ^††^P < 0.01 versus 1D 23 ℃, Dunnett test, n = 4 per group).

To investigate the storage stability of the growth factors contained in the dry sheets, we examined the storage temperatures and storage durations using dry sheets prepared simultaneously. Dry sheets were stored at refrigeration temperature (4 °C) and room temperature (23 °C) for 1 day and 1, 2, or 4 weeks. Next, the eluates were prepared by immersing each dry sheet in the medium for 24 h and stored at − 30 °C until measurement. The concentrations of VEGF and HGF did not show major fluctuations with temperature or storage period, but the concentrations of FGF-2 and HMGB1 gradually decreased at room temperature during the 4-week storage period (Fig. 7). These results demonstrate that refrigerated storage is suitable for dry sheets and that they remain stable for at least 4 weeks.

## Discussion

In cell sheet transplantation therapy, it is theorized that the most important effectors are living cells, and that therapeutic effects can be obtained due to the factors they produce. In accordance with this theory, previous studies have shown that cell sheets produce various growth factors and cytokines, including VEGF, HGF, transforming growth factor-beta1, and monocyte chemotactic protein 1, which promote wound healing^7,9^. Therefore, it is generally accepted that nonviable dry sheets do not continuously secrete growth factors and can only promote wound healing to a limited extent. Surprisingly, our data demonstrated that dry sheets using autologous or allogeneic cells were not significantly less effective than living sheets and exerted a significant promotion of wound healing compared to the no-treatment control group (Fig. 5). This was likely induced by the bioactive substances released from the dry sheets, given the differences between dry and FT sheets.

We found that similar amounts of growth factors and cytokines, including VEGF, HGF, FGF-2, and HMGB1, were preserved in cells of dry sheets as in those of living sheets, and that these substances were readily released from cells into the solution (Fig. 3a,b). Notably, FGF-2 in the cytoplasm and HMGB1 in the nucleus were not secreted from the living sheets but were released from the dry sheets (Fig. 3b). We confirmed that the dry sheet eluate exhibited stronger bioactivity than living sheet eluate by in vitro examination of a fibroblast proliferation assay (Fig. 4a). In addition, the eluate stimulated fibroblasts and enhanced VEGF and HGF production (Fig. 4b–d), suggesting that substances released from the dry sheets promote, both directly and indirectly, wound healing and angiogenesis.

High levels of FGF-2 were released from the dry sheets into the medium and were involved in biological activity. FGF-2 is a potent growth factor and important for promoting wound healing and angiogenesis^16^. Although FGF-2 is highly expressed in fibroblasts, its extracellular secretion is generally inefficient due to the lack of secretory signal peptides. Therefore, the major FGF-2 release is from damaged or dead cells^17–20^. A previous study on mesenchymal stromal cell (MSC) transplantation therapy showed that high levels of FGF-2 released from injured transplanted MSCs stimulated angiogenesis and neuropoiesis^20^. Subsequently, there have been no reports on FGF-2 as an effector secreted from cell sheets. In this study, we demonstrated that the dry sheet eluate promoted the proliferative activity of fibroblasts, which was markedly suppressed by the FGF-2 neutralizing antibody (Fig. 4f). These findings suggest that the release of FGF-2 from dry sheets plays an important role in promoting wound healing and angiogenesis.

**Figure 8:**
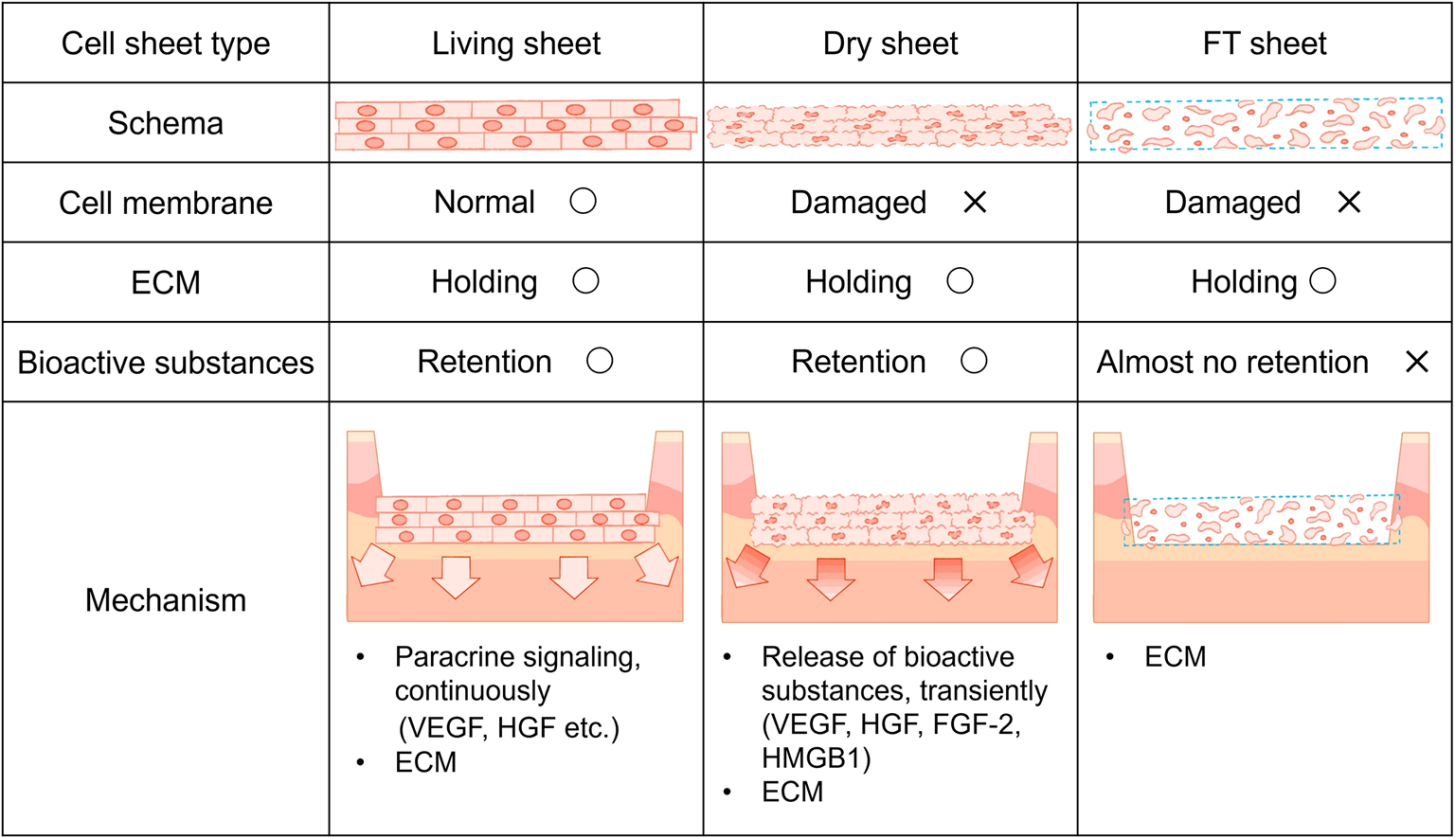
Schema of the mechanism for wound healing effect of each cell sheet. Living sheets were considered to have the greatest therapeutic effect by engrafting after application to the wound. While the living sheets were alive, the paracrine effect of continuously secreting large amounts of growth factors promoted wound healing. When dry sheets are transplanted into wounds, they transiently release various growth factors, which are thought to stimulate angiogenesis and the activation of cells around the wound. In addition, all cell sheets retained ECM, which may have promoted wound healing by protecting the wound. Owing to these differences in the mechanism of action, the ranking of the acceleration of wound healing was as follows: living sheets > dry sheets > FT sheets. ECM: extracellular matrix.

The in vivo experiment using a diabetic mouse skin wound model showed that there was no difference in wound closure between the dry sheet group and control group on day 13. However, there was a significant difference in the rate of wound closure by macroscopic observation at 5, 7, and 9 days after treatment (Fig. 5). On days 5 and 7 after treatment, histologically, epithelialization significantly increased in the dry sheet group (Supplementary Fig. S6e–h), and there was a significant difference in the number of microvessels and keratinocyte growth at the wound edge on day 3 after treatment (Supplementary Fig. S6a–d). These findings indicate that growth factors were released from the dry sheets during the early phase after transplantation, and that they stimulated both fibroblasts and peri-wound cells, including keratinocytes and vascular endothelial cells, both directly and indirectly, which may have promoted wound healing. We observed that the sheet-like structure containing ECM seemed to protect the wound from external factors (Fig. 6), but we could not confirm cell proliferation inside the sheet structure. The characteristics and possible mechanisms of action of the cell sheets used in this study are summarized in Fig. 8.

When the dry sheet eluate was treated with an excess amount of the FGF-2 neutralizing antibody, cell proliferation was inhibited to the same level as that of the control. However, the cell proliferative response to the dry sheet eluate was significantly higher than that to rFGF-2 alone (Fig. 4f). This result indicated that substances other than FGF-2 may also affect cell proliferation. Although further studies are needed, nuclear substances, such as HMGB1, which are collectively known as alarmins and act as defense factors^21,22^ and are involved in promoting wound healing^23–26^, are also released from the dry sheets, suggesting that multiple factors may act in concert to promote wound healing.

In our previous study, living sheets transplanted into mouse cutaneous ulcer models, both autologous and allogeneic, disappeared by day 9 of in vivo imaging of luciferase-expressing fibroblasts^9^. Similarly, histological observations of the wound healing process indicated that the allogeneic dry sheets were located under the epidermal layer during epithelialization, but no dry sheets remained in the tissue specimens after the completion of epithelialization on day 9 (Fig. 6). It was suggested that the dry sheet naturally decomposed and was absorbed by the infiltrating leukocytes. A previous study using human-derived epidermal cell sheets in a mouse ulcer model assuming allogeneic transplantation in humans confirmed that the sheets were excreted ex vivo during the wound healing process^27^. These observations suggest that fibroblast-derived dry sheets can be used not only on the body surface but also on internal organs because they are decomposed and absorbed. Previously, we have also demonstrated the effectiveness of a multi-layered fibroblast cell sheet for postoperative bronchial and pancreatic fistulas in animal models^28,29^. Further, dry sheets can also be used as biological coating materials to promote tissue repair and prevent various postoperative complications.

Here, we confirm that the bioactive substances retained in the refrigerated dry sheet were stable for at least 1 month and that some bioactive substances were retained even when stored at room temperature (Fig. 7). Although dry sheets stored at room temperature for 7 days or less were used in the mouse experiments in this study, the dry sheet treatment group showed significant wound healing promotion effects compared with the no-treatment group. Since the in vitro storage stability study revealed that the amount of FGF-2 is affected by the storage temperature, a better therapeutic effect may be expected by strictly controlling the storage temperature.

Dry sheets maintain their sheet-like structure while retaining a certain degree of rigidity, meaning they can be used without the support required for cell sheet transplantation therapy and have the advantage of easier handling (Supplementary Fig. S1). Several dehydrated amniotic membrane sheets have already been used for the treatment of skin ulcers^30^. Fibroblast-derived dry sheets have advantages in terms of stability of material availability and safety from infections caused by donors. Our dry sheets can be mass-produced from a single material, ensuring a stable supply and uniform product quality.

As a limitation, following our previous studies^5–7^, all cell sheets used in this study were produced from hypoxic preconditioned multi-layered fibroblast cell sheets since the treatment of hypoxic preconditioning is effective in increasing the secretory potency of the fibroblast. Before this study, we hypothesized that dry sheets could provide wound healing effects through the same growth factors secreted by living sheets. However, unlike the mechanism of action of conventional cell sheets, it became clear that the release of FGF-2 from dry sheets, which is not secreted by living sheets, plays an important role in wound healing. Therefore, the effect of hypoxic preconditioning on growth factors retained in the dry sheet needs to be investigated in the future.

In conclusion, this is the first study to demonstrate that the wound healing ability of allogeneic dry sheets for cutaneous ulcers is comparable to autologous dry sheets. Importantly, we found that the dry sheets retained and released bioactive substances, including FGF-2, from the cells to promote wound healing. Dry sheets can be easily refrigerated or stored at room temperature and can be quickly and stably supplied. Thus, allogeneic dry sheets represent a useful tool for treating cutaneous ulcers.

## Material and methods

### Ethics declaration

All experiments in this study were performed in accordance with the relevant guidelines and regulations. All animal procedures were carried out in compliance with the ARRIVE guidelines and approved by the Institutional Animal Care and Use Committee of Yamaguchi University (approval number 31-093).

### Animals

Male C57BL/6N and C3H/He mice (6-weeks old) were purchased from Japan SLC (Shizuoka, Japan). They were housed in a temperature—(22 ± 2 °C), humidity—(70 ± 20%), and light—controlled room (12-h light/dark cycles) with ad libitum access to food and water.

### Preparation of cell sheets

#### Multi-layered fibroblast cell sheets (living sheets)

Fibroblasts were isolated from the tail skin of mice using collagenase (FUJIFILM Wako Pure Chemical Corporation, Osaka, Japan) and cultured in CTS™ AIM-V™ medium (Thermo Fisher Scientific, Waltham, MA, USA) with 10% fetal bovine serum (FBS; Thermo Fisher Scientific). Primary fibroblast cells were seeded in a normal 24-well plate (4.2 × 10^5^ cells/well) using 2 mL medium consisting of CTS™ AIM-V™ and HFDM-1 (+) (Cell Science & Technology Institute, Sendai, Japan) supplemented with 5% FBS and were incubated under normoxic conditions (37 °C, 5% CO_2_, 20% O_2_) for 2 days, followed by hypoxic conditions (33 °C, 5% CO_2_, 2% O_2_) for 1 day for the treatment of hypoxic preconditioning^5–7^. After incubation, multi-layered fibroblast cell sheets were rinsed twice with 2 mL phosphate-buffered saline (PBS) and then incubated with 500 µL dispase solution (10 PU/mL, FUJIFILM Wako) for 30 min under normoxic conditions. After washing twice with PBS, multi-layered fibroblast cell sheets were gently detached from the culture plates. These sheets were used as the living sheets in this study.

#### Dried cell sheets (dry sheets)

Air-drying was performed inside a bio-clean bench (CCV-1300E, Hitachi, Ltd., Tokyo, Japan), with the pilot flame of the gas burner operating in the center, where clean operations could be maintained. Living sheets were transferred onto a silicon pedestal using a 1000 µL wide-bore tip and unfolded to avoid wrinkling. After removing as much water as possible, the sheets were left to stand for 30 min. The dried cell sheets were peeled off from the silicon with tweezers and transferred to 1.5-mL microtubes. For transplantation experiments, dry sheets were stored at room temperature (23 °C) and used within 1 week after preparation. For storage stability experiments, dry sheets were stored with a desiccant in a refrigeration temperature (4 °C) or room temperature (23 °C).

#### Freeze–thaw (FT) cell sheets

A 24-well culture plate containing living sheets was transferred to a sealable plastic bag and placed in a deep freezer (− 80 °C) for 60 min, followed by thawing in an incubator (37 °C) for 90 min. The freeze and thaw cycles were repeated three times to destroy cell membranes, followed by washing twice with 2 mL PBS to obtain FT sheets. FT sheets were stored after the third freezing cycle (− 80 °C) until use.

### Measurement of the rate of drying, weight, and moisture content in dry sheets

Eight living sheets were transferred onto a plastic plate using a 1000 µL wide-bore tip, and the weight change was measured every minute using a balance XS104 (Mettler Toledo, Inc.) installed on a bio-clean bench. Three independent experiments were performed and the drying speed was calculated. The environmental conditions in the bio-clean bench were measured by using HYGROPALM-HP32 (Rotronic, Bassersdorf, Switzerland) and INFURIDER Handy Anemometer (AP-816B, AOPUTTRIVER).

The total weight of the 50 dry sheets was measured, and the sheets were immersed in 2 mL methanol for two hours. The amount of water in the methanol was measured using the Karl Fischer method (Japan Testing Laboratories, Inc., Ohgaki, Japan). The Karl Fischer method was performed twice.

### DAPI staining of unfixed cell sheets

Unfixed cell sheets were stained with DAPI (Dojindo, Kumamoto, Japan). Images were captured using a BZ-X710 microscope (Keyence, Osaka, Japan).

### LDH release assay

Individual living sheets were air-dried for 5, 10, 15, 30, 45, or 60 min and immediately immersed in 500 µL PBS for 30 min at room temperature. The LDH in the supernatant of each cell sheet was measured with the living cell sheet immersed in 500 µL cell lysis buffer as the positive control and PBS as the negative control using an LDH Cytotoxicity Detection Kit (Dojindo).

### Evaluation of the proliferative capacity and cellular metabolic activity of re-cultured cell sheets

The living sheets and dry sheets obtained after drying for 30 min were transferred to another 12-well plate containing 2 mL CTS™ AIM-V™ with 10% FBS and cultured for 24 h under normoxic conditions. The cell sheets were observed using a phase-difference optical microscope. The cellular metabolic activity of the re-cultured cell sheets was analyzed as follows: after removing the culture supernatants, 1 mL CTS™ AIM-V™ with 10% FBS containing 10% [2-(2-methoxy-4-nitrophenyl)-3-(4-nitrophenyl)-5-(2,4-disulfophenyl)-2H-tetrazolium, monosodium salt] (WST-8) reagent (Cell Count Reagent SF; Nacalai Tesque, Inc. Kyoto, Japan) were added to each well and incubated for 3 h under normoxic conditions. The absorbance of the supernatant was measured at 450 nm (reference wavelength: 630 nm).

### Histological analysis and immunostaining

The detailed protocol was described previously^7^. In brief, cell sheets were transferred onto a CellShifter (CellSeed Inc., Tokyo, Japan) to prepare tissue sections. Isolated cutaneous tissues or cell sheets were fixed in a 10% formalin neutral buffer solution and embedded in paraffin. Three-micrometer-thick sections were mounted on slides and stained with hematoxylin–eosin (HE) or Azan. Immunostaining was performed using primary rabbit antibodies for anti-collagen I (1:200, ab34710, Abcam, Cambridge, UK), anti-FGF-2 (1:30, ab208687, Abcam), and anti-HMGB-1 (1:400, ab79823, Abcam), rabbit anti-CD31 antibody (1:200, ab28364, Abcam) and a secondary antibody for DyLight550 conjugated goat anti-rabbit IgG (H&L) (1:200, ab96884; Abcam). DAPI was used to stain the cell nuclei. All histological images were captured by BZ-X710 microscope and analyzed using BZ-X analyzer (Keyence). All histological analyses were performed by a pathologist.

### Enzyme-linked immunosorbent assay (ELISA)

Cell sheets were immersed in 1.5 mL microtubes with their lid sealed. To measure cytokine levels inside the cell sheets, each cell sheet was immersed in 200 μL Cell Lysis Buffer 2 (R&D Systems, Inc. Minneapolis, MN, USA) for 30 min at room temperature. To measure cytokine levels released from the cell sheets, each cell sheet was immersed in 200 µL CTS™ AIM-V™ with 10% FBS for 24 h under normoxic conditions (37 °C). After incubation, samples were centrifuged at 2460×*g* for 5 min at 4 °C, and supernatants were collected and stored at − 30 °C until measurement. The concentrations of VEGF, HGF, FGF-2, and HMGB1 in the supernatant were measured using Quantikine Immunoassay Kits (R&D Systems) and HMGB1 ELISA Kit Exp (SHINO-TEST CORPORATION, Kanagawa, Japan), respectively, according to the manufacturer’s instructions.

### Cell proliferation, VEGF and HGF production analysis in fibroblasts following incubation with eluate from cell sheets

The eluate samples were prepared by immersing each cell sheet in 200 μL DMEM (Thermo Fisher Scientific) without FBS for 24 h under normoxic conditions. The supernatant was collected after centrifugation (2460×*g*, 4 °C, 5 min). Fibroblasts were seeded in 96-well plates at 8000 cells/well in 100 μL DMEM with 10% FBS, followed by addition of 100 μL of either the eluate sample or DMEM without FBS. Each eluate sample diluted 2-folds with DMEM containing 10% FBS was used for as an eluate sample without fibroblasts. After 48 h of incubation under normoxic conditions, the culture supernatant was collected for the measurement of VEGF and HGF, followed by the cell proliferation assay. DMEM (100 μL) with 5% FBS containing 10% WST-8 reagent (Cell Count Reagent SF) was added to each well and incubated for 1 h under normoxic conditions. The absorbance of the supernatant was measured at 450 nm. The cell proliferation rate was calculated using the fibroblasts cultured in DMEM as the control. The concentrations of VEGF and HGF in the culture supernatant were measured by ELISA, and the production ratios were calculated by following formula: [(supernatant of fibroblasts cultured with the eluate sample) − (supernatant of the eluate sample without fibroblasts)]/(supernatant of fibroblasts cultured with DMEM). Three independent experiments were performed in triplicate.

### Neutralizing antibody experiment

Dry sheet eluate samples were prepared by the same procedure as for the cell proliferation analysis described above. DMEM containing rFGF-2 (0 or 5 ng/mL, FUJIFILM Wako) were also prepared. These samples were incubated for 60 min at 37 °C with the anti-FGF-2 neutralizing antibody (30.3 μg/mL, #05-117, clone bFM-1, Merck Millipore, Darmstadt, Germany), or the control antibody (Mouse IgG1 isotype control, clone11711; 30.3 μg/mL, MBA002, R&D Systems). Fibroblasts were seeded in 96-well plates at 8000 cells per well in 100 μL DMEM with 1% FBS, followed by the addition of 100 μL of either the eluate sample with the antibodies, or DMEM containing rFGF-2 (0 or 5 ng/mL) with antibodies. After a 48 h incubation under normoxic conditions, the culture supernatant was aspirated and the cell proliferation assay was performed. DMEM (100 μL) with 0.5% FBS containing 10% Cell Count Reagent SF was added to each well and incubated for 2 h under normoxic conditions. The absorbance of the supernatant was measured at 450 nm. The cell proliferation rate was calculated using fibroblasts cultured in 0 ng/mL rFGF-2 with the control antibody as the control. Three independent experiments were performed in triplicate.

### Mouse cutaneous ulcer model and cell sheet transplantation

Male C57BL/6N mice were intraperitoneally administered streptozotocin (STZ; FUJIFILM Wako) at 55 mg/kg every 24 h for five consecutive days. Mice with blood glucose values of ≥ 300 mg/dL on days 9 and 10 after administration of STZ were classified as diabetic mice. A skin ulcer was prepared in the diabetic mice on the 14th day after the last administration of STZ. Male C57BL/6N mice were anaesthetized with 2% isoflurane via inhalation, and a 6 mm full-thickness skin defect was created on the dorsal skin using a biopsy punch (n = 6 per group). Living sheets and FT sheets were transferred onto the skin defect using a 1000 µL wide-bore tip, and dry sheets were handled using tweezers. Each cell sheet was prepared from male C57BL/6N mice (autologous) and male C3H/He mice (allogeneic). All wounds were covered with ADAPTIC (#2012; Acelity, San Antonio, TX, USA) and Derma-aid^®^ (ALCARE, Tokyo, Japan) and fixed with Silkytex bandage (#11893; ALCARE) for the first 24 h. On the first day, all wounds were covered with Airwall Fuwari (# MA-E050-FT; Kyowa, Osaka, Japan) and fixed using Silkytex bandage^9^. Each wound was photographed using a digital camera on days 0,1, 3, 5, 7, 9, 11, and 13. Each photograph was normalized with a 10.5-mm diameter measurement, and the wound area was measured by manually tracing each wound edge using ImageJ software (NIH, Bethesda, MD, USA). The wound closure rate was calculated as follows: [day X] (%) = {1 − (wound area [day X]/wound area [day 0])} × 100. Dry sheets were stored at room temperature (23 °C) and used for this experiment within 1 week after preparation.

### Statistical analysis

Results are presented as mean ± standard deviation, unless indicated otherwise. Statistical differences were assessed by one-way analysis of variance followed by either the Tukey–Kramer test for multiple comparisons between groups or the Dunnett test for comparisons of multiple groups against a control group. Student’s *t*-test was performed for a statistical comparison between the two groups. Data were statistically analyzed in Statcel (Add-in software for Microsoft Excel, OMS Ltd., Japan). Statistical significance was set at *P* < 0.05.

## Supplementary Information

Supplementary Figures.

## Data Availability

The datasets generated and analyzed during the current study are available from the corresponding author upon reasonable request.
