# Supplementary material for: Dry preserved multilayered fibroblast cell sheets are a new manageable tool for regenerative medicine to promote wound healing
Source: Sci Rep. 2022 Jul 22;12:12519. doi: 10.1038/s41598-022-16345-6 (PMC9307603; doi:10.1038/s41598-022-16345-6)
Supplement: Supplementary file 1 — Supplementary Figures. [file 41598_2022_16345_MOESM1_ESM.pdf]

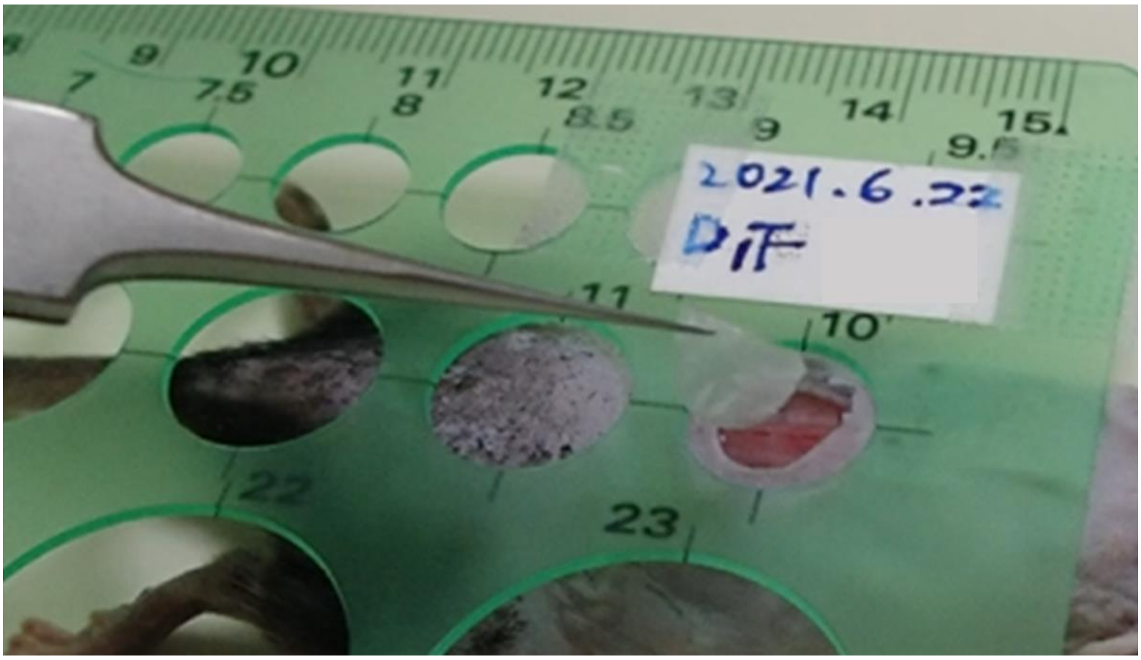

Supplementary Figure S1. Photograph of dry sheet transplantation to the wound in back skin of a mouse.

The dry sheet was easily handled, grasped and transplanted with tweezers.

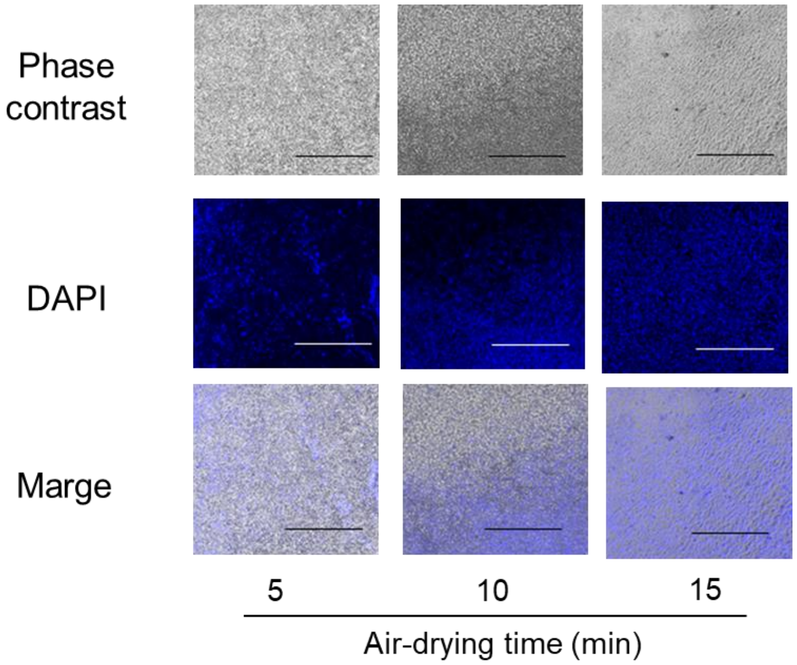

Supplementary Figure S2. Cell sheets stained with DAPI over drying time.

At drying times of 5, 10, and 15 min, the nuclei of cell sheets were stained with DAPI from the cell sheet periphery, but there were unstained areas in the center of the cell sheets.

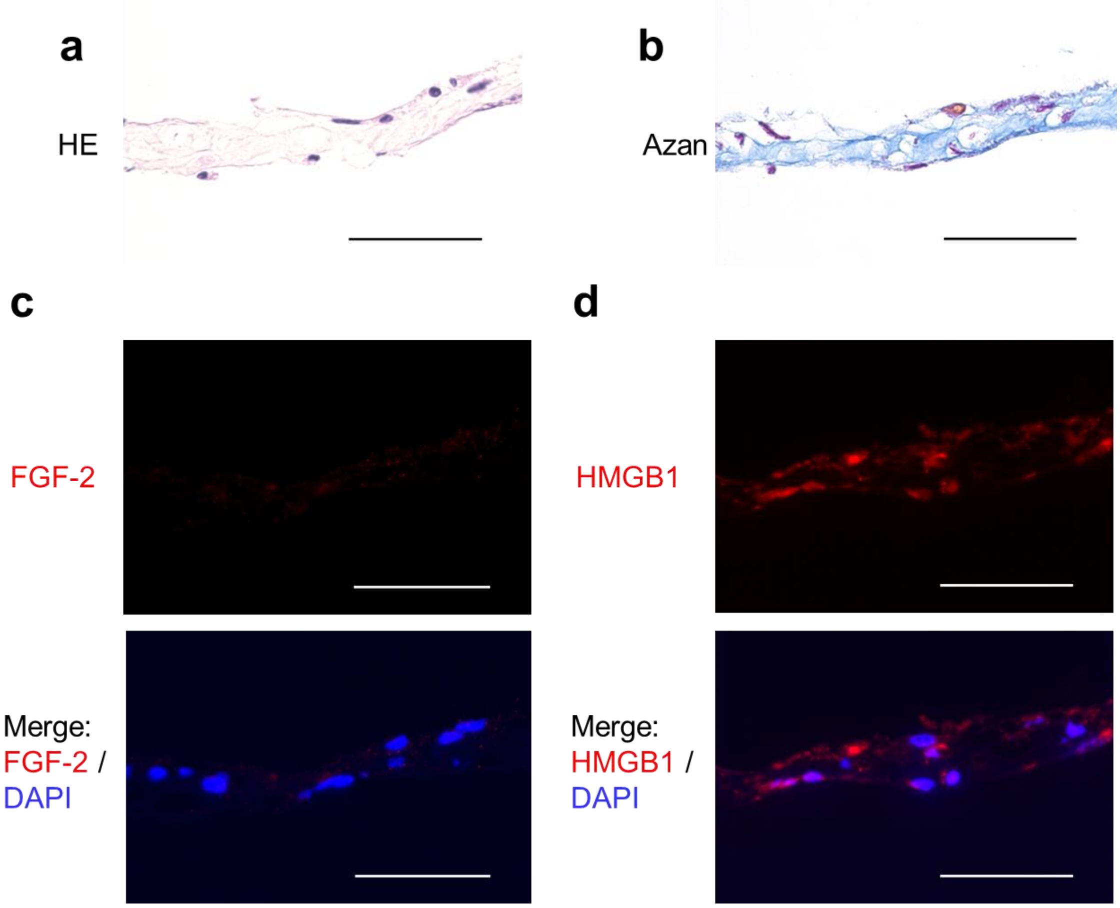

Supplementary Figure S3. Histological analysis of the dry sheet.

(**a**) Cross sections of the dry sheet with HE staining (scale bar = 50 μm). (**b**) Cross sections of the dry sheet with Azan staining (scale bar = 50 μm). (**c**) Cross-sections of the dry sheet with immunofluorescence staining of FGF-2 (red) and DAPI (blue; scale bar = 50 μm). (**d**) Cross-sections of the dry sheet with immunofluorescence staining of HMGB1 (red) and DAPI (blue; scale bar = 50 μm).

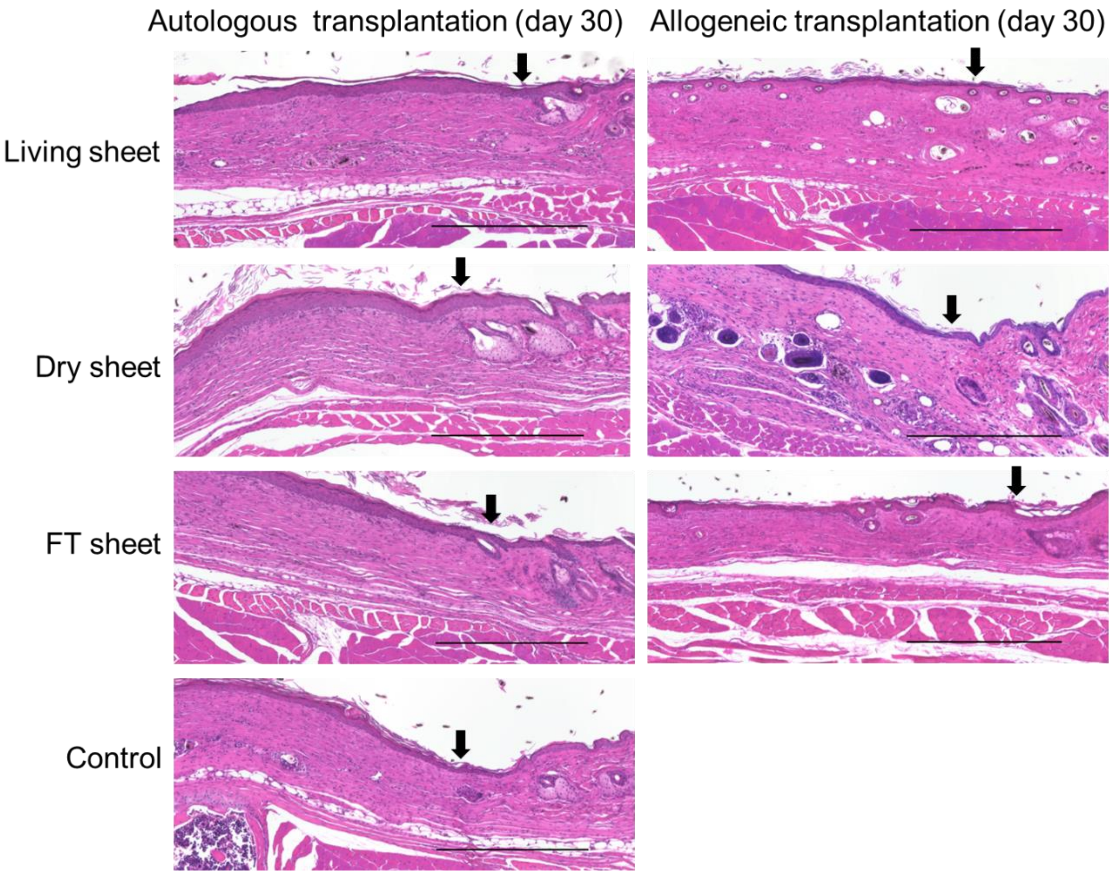

Supplementary Figure S4. Histological analysis 30 days after transplantation of each cell sheet.

Representative cross-sections of HE staining 30 days after transplantation (scale bar = 500 µm).

Black arrows indicate the scar edges.

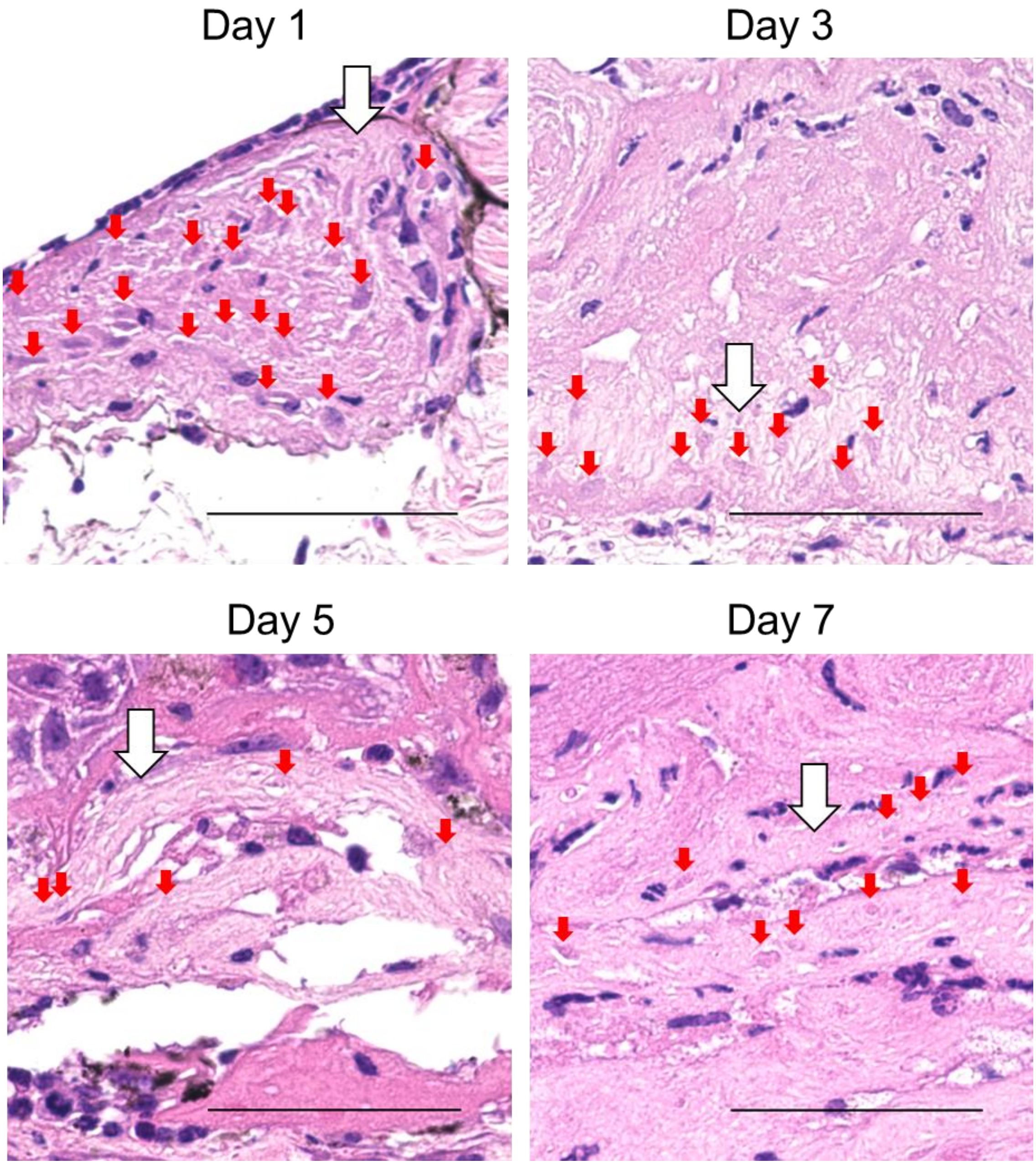

Supplementary Figure S5. Phagocytosis of the allogeneic dry sheet.

HE-stained cross-section of a mouse cutaneous wound transplanted with the dry sheet on days 1, 3, 5, and 7 (scale bar = 50 μm). White arrows indicate dry sheets. The red arrows indicate the nuclei of dry sheet-derived cells.

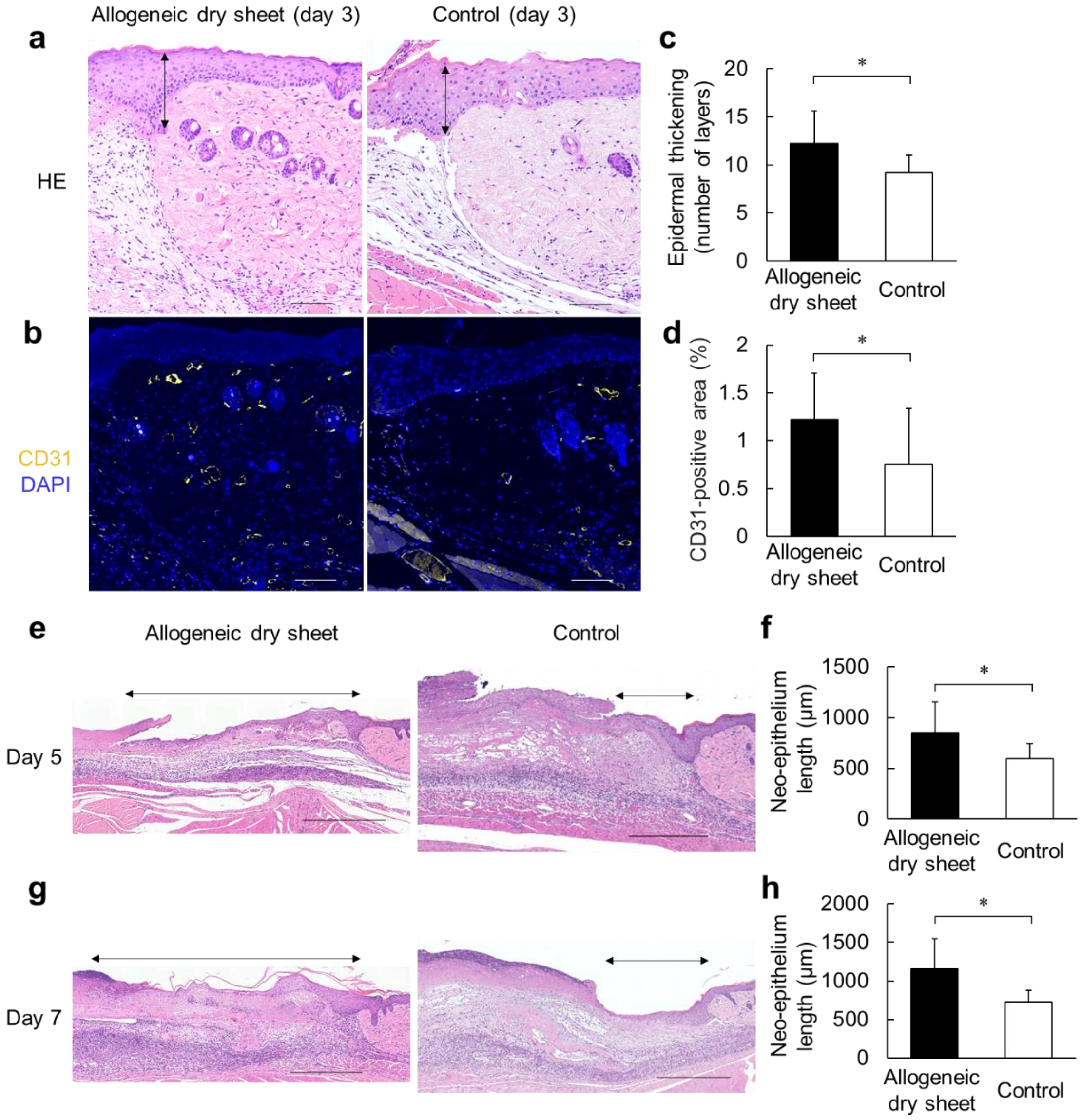

Supplementary Figure S6. Histological analysis of wound edge after transplantation.

(a) Representative cross-section of HE-stained wound edge on day 3 after transplantation (scale bar = 50 μm). Black arrows indicate the measurement site of the keratinocyte layer at the wound edge. (b) Representative cross-sections of immunofluorescence-stained wound edge on day 3 after transplantation with CD31 (yellow) and DAPI (blue); (scale bar = 50 μm). (c) Number of layers of keratinocytes in the wound edge (n = 3 per group, four wound edges per one cross-

section). Values are expressed as mean ± SD (*: $P < 0.05$, Student t-test). (**d**) The area percentage of CD31-positive cells in the dermal wound at the wound edge was calculated (n = 3 per group, four wound edges per one cross-section). Values are expressed as mean ± SD (*: $P < 0.05$, Student t-test). (**e**) Representative cross-section of HE-staining on day 5 after transplantation (scale bar = 500 µm). Black arrows indicate neo-epidermis. (f) Neo-epidermis length on day 5 after transplantation (n = 2 per group, four wound edges per one cross-section). Values are expressed as mean ± SD (*: $P < 0.05$, Student t-test). (**g**) Representative cross-section of HE-staining on day 7 after transplantation (scale bar = 500 µm). Black arrows indicate neo-epidermis. (h) Neo-epidermis length on day 7 after transplantation (n = 2 per group, four wound edges per one cross-section). Values are expressed as mean ± SD (*: $P < 0.05$, Student t-test).
